# Partial Anterior Opercular Syndrome as Surgical Complication: Case Presentation and Brief Review of Literature

**DOI:** 10.1155/crnm/9136610

**Published:** 2025-09-24

**Authors:** Francesco Salomi, Erika Ferrari, Pietro Zangrossi, Lorenzo Tinti, Michele Terzaghi, Francesco Guerrini, Giannantonio Spena

**Affiliations:** ^1^Neurosurgery Unit, Head and Neck Department, Fondazione IRCCS Policlinico San Matteo, Pavia, Italy; ^2^Department of Neurosurgery, Sant'Anna University Hospital, Ferrara, Italy; ^3^Neurology Unit, Fondazione IRCCS Mondino Neurological Hospital, Pavia, Italy

## Abstract

Anterior opercular syndrome (a.k.a. Foix–Chavany–Marie syndrome) is a rare neurological condition, described as a paralysis of the mouth and tongue usually caused by a bilateral lesion of the frontal opercular area. The patient presents with speaking, chewing, and swallowing impairment, but autonomic and emotional functions—like smiling and yawning—are typically preserved. We present our patient's clinical data after critical analysis, together with a brief literature review about anterior opercular syndrome caused by unilateral opercular lesions. To our knowledge, less than 20 cases of anterior opercular syndrome caused by unilateral lesions are described in the literature. In some patients, a contralateral lesion can be detected on brain imaging in regions different from the anterior opercular cortex. This syndrome can rarely occur as a consequence of unilateral opercular cortex damage. The possible role of contralateral lesions located in neuronal pathways functionally related to the anterior operculum requires further investigation.

## 1. Introduction

The anterior opercular syndrome, also known as Foix–Chavany–Marie (FCM) syndrome, manifests as a rare form of pseudobulbar palsy affecting the lower cranial nerves. It is characterized by pronounced dysarthria or anarthria and dysphagia, coupled with bilateral central paralysis affecting facial, pharyngeal, lingual, and masticatory muscles. Patients typically present with a sudden onset of facial flaccidity, speech impairments, and significant difficulties in chewing and swallowing (during the oral phase). In some instances, there may be an exaggerated jaw reflex leading to the development of trismus. An indicative feature of the syndrome is an automatic-voluntary dissociation, allowing for spontaneous expressions like smiling, crying, or yawning [1].

This syndrome can manifest at any age. In children, it may arise due to congenital factors such as polymicrogyria or as a consequence of acquired conditions like encephalitis, neurodegenerative diseases, or trauma. It often coincides with delays in achieving developmental milestones and may be accompanied by epilepsy [2, 3]; albeit rare, familial occurrences have been reported. Among adults, common etiologies include ischemic events, infections, traumatic injuries, neoplasms, and epileptic seizures. To our knowledge, there are approximately 200 documented cases in medical literature, with bilateral lesions of the frontal opercular cortex typically implicated as the underlying cause. In this paper we present the case of a rare unilateral opercular syndrome and the first case described in the literature to have occurred following surgery in a patient with a concomitant pontine lesion.

## 2. Case Presentation

A 43-year-old man was admitted to our neurological department reporting lumbar arthralgia, right leg pain, and mild muscle weakness. Upon neurological examination, he exhibited paralysis of the left arm and forearm, limited finger mobility, weakness in the right lower limb, and deviation of the oral rim to the left. A swallowing assessment revealed mild oropharyngeal muscles paralysis. No speech disorder was detected.

His medical history included a diagnosis of ankylosing spondylitis, confirmed by positive Human Leukocyte Antigen (HLA)-B27. In the six months prior to hospitalization, he had undergone intravenous antibiotic therapy (imipenem and linezolid) for suspected cerebral nocardiosis, with subsequent clinical and radiological improvement. Additionally, he had a biopsy of a palmar lesion, which indicated lobular and septal panniculitis.

**Figure 1:**
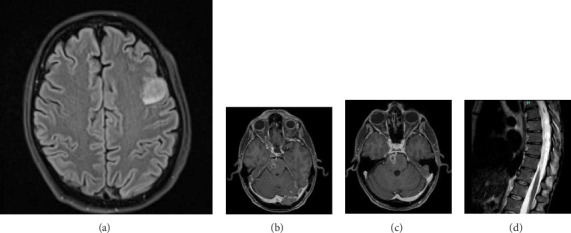
(a) Preoperative magnetic resonance imaging showing a hyperintense lesion in the left frontal opercular region on FLAIR sequences. (b and c) In contrast-enhanced sequences, a right paramedian pontine lesion involving the trigeminal nerve and Meckel's cave was evident. (d) The spine MRI has also revealed a signal of spinal cord hyperintensity from T3 to the conus.

Brain magnetic resonance imaging (MRI) revealed two contrast-enhancing lesions at the cortical-subcortical junction: one in the right posterior temporal region and another in the left anterior frontal region. During his hospital stay, he experienced generalized tonic-clonic seizures and flaccid paralysis of the lower limbs, accompanied by fever spikes. A subsequent brain and spine MRI showed a new hyperintense lesion in the left middle frontal gyrus on FLAIR sequences (Figure 1(a)), as well as pathological enhancement along the right ventral pontine rim (Figure 1(b)) and towards the right Meckel's cave (Figure 1(c)). Furthermore, there was suspicion of myelitis affecting the spinal cord from T3 to T12 (Figure 1(d)).

Despite extensive blood and cerebrospinal fluid tests, no significant abnormalities were found, ruling out systemic spread of infectious disease. After multidisciplinary consultation, a decision was made to perform a cerebral biopsy targeting the largest, most accessible lesion in the left frontal region. Despite being informed of the risks, the patient consented to the procedure.

**Figure 2:**
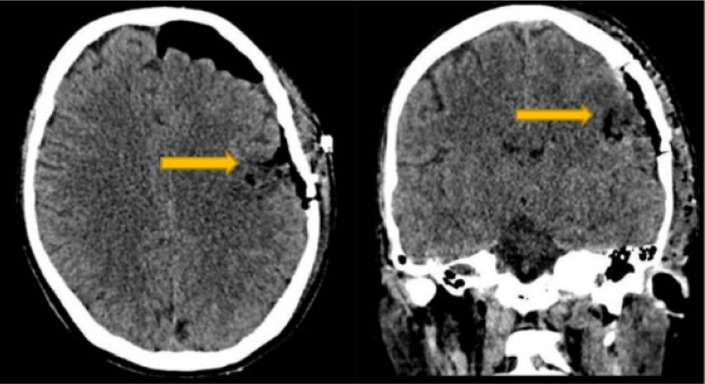
The postoperative brain CT scan ruled out the presence of postsurgical acute complications.

**Figure 3:**
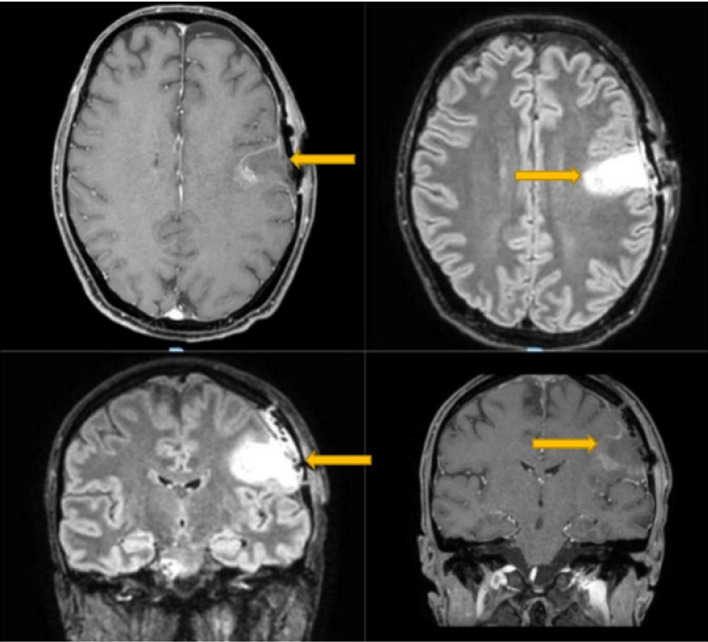
The postoperative magnetic resonance imaging did not show any new onset lesions near the surgical site or in the right operculum or in the subcortical fibers.

Upon awakening from the biopsy, the patient exhibited trismus (clenched jaw) and paralysis of facial muscles with an inability to voluntarily open his mouth or move his tongue and a consequent anarthria. Classical causes such as drug side effects, tetanus, or infections were ruled out, as blood tests, cultures, and a lumbar puncture for cerebrospinal fluid analysis were repeated, all of which were within normal limits. Acute lesions in the contralateral opercular area were ruled out through radiological imaging; both head CT and MRI showed the presence of the surgical cavity (yellow arrows), without any ischemic or hemorrhagic lesions (Figures 2 and 3). The patient was still able to sleep with his mouth open and with involuntary facial movements present. A swallowing assessment showed paralysis of pharyngeal and laryngeal muscles, so a nasogastric tube was inserted for medication and feeding, and oral carbamazepine and baclofen were initiated in order to modulate muscle tone. Over 3 months, there was gradual improvement in trismus, although anarthria persisted. Histological analysis of the biopsy revealed nonspecific inflammatory tissue.

## 3. Discussion

Anterior opercular syndrome, a rare condition typically arising from bilateral frontal opercular damage, manifests with distinct neurological signs including bilateral paralysis of cranial nerves V, VII, IX, X, and XII, leading to evident dysphagia, anarthria, and palsy of the mouth and tongue. A notable feature is the automatic-voluntary dissociation, preserving movements with autonomic and emotional components, attributed to the involvement of the limbic system and its connections with the brainstem [4].

Only 14 documented cases in literature depict this syndrome resulting from unilateral opercular lesions (Table 1), with most exhibiting a contralateral lesion on MRI scans, albeit located in regions other than the frontal operculum. No clear correlation between lesion side and dexterity has been established.

Complex functions such as facial movements and swallowing necessitate bilateral coordination mediated by cortical-ponto-cerebellar networks. In a case described by Sá et al., a patient with left opercular infarction and an old ischemic lesion of the right cerebellum suggested a potential causal link, as the cerebellum also contributes to motor control [13]. Similarly, Torres-Perales et al. hypothesized cerebellar involvement due to its role in adjusting motor expression to emotional contexts [17]. Recent reports, like that of Rivas et al., have suggested a connection between opercular infarction and bilateral subcortical vascular encephalopathy [1].

The occurrence of this syndrome with unilateral opercular lesions may stem from contralateral disruption of corticobulbar fibers or hemispheric dominance in controlling cranial nerve nuclei bilaterally [4, 14]. Post-surgical damage to the right opercular area without MRI-detectable lesions has been reported [11], as well as rare cases where cerebral lesions are not apparent on MRI but are revealed through brain SPECT, affecting cortical-ponto-cerebellar pathways [18, 19].

In our patient, alongside the left opercular lesion, involvement of the right paramedian pons, the right trigeminal nerve, and Meckel's cave was noted. Trismus, or hypertonicity of chewing and perioral muscles, may result from bilateral upper motor neuron damage: one at the cortical level (left) and one along the cortico-bulbar fascicles (right).

Our patient did not exhibit a pure autonomic-voluntary dissociation, possibly due to interruption of the connection between the amygdala and brainstem caused by the pontine lesion [14]. Interestingly, symptom relief occurred only during sleep, likely attributable to the well-known loss of muscle tone during “rapid eye movement” (REM) sleep mediated by the sublaterodorsal tegmental nucleus in the dorsal rostral pons, leading to glycinergic inhibition of orofacial muscles [20].

Prognosis and natural history vary based on the underlying cause. Only one case reported near-complete recovery after surgical drainage of a left temporal operculum abscess [16]. Clinical improvement is rare and often partial, with onset observed from 2 weeks to several months postsymptom onset [1, 21]. Supportive therapies and rehabilitation are mainstays of treatment, with acute-phase risks primarily related to aspiration and subsequent pneumonia necessitating nutritional support via nasogastric tube or percutaneous endoscopic gastrostomy. While dysphagia, mouth, and tongue paralysis may show a better prognosis, anarthria tends to persist, with minimal improvement over time.

Anterior opercular syndrome commonly results from bilateral frontal opercular lesions. Nonetheless, there are documented instances where a unilateral opercular lesion coincides with contralateral lesions affecting neural circuits responsible for mouth and tongue movements [18, 19]. Consequently, a comprehensive imaging evaluation, including MRI and advanced techniques such as SPECT, is crucial for assessing these patients, particularly prior to surgical interventions. Given the ongoing quest to fully elucidate the pathophysiological mechanism driving this syndrome, our current report contributes additional hypotheses regarding potential mechanisms.

## Figures and Tables

**Table 1:** Summary of literature review of anterior opercular syndrome with unilateral opercular lesion.

Reference	Age	Sex	H	Cause	Side	Site	Concomitant lesions	Symptoms	Outcome
Cosnett et al. (1988) [5]	67 yo	M	L	Abscess	L	Frontal operculum	None	Anarthria, facial diplegia, dysphagia	Improvement of dysphagia and tongue palsy, persistent dysarthria at 2 years
Bakar et al. (1998) [6]	39 yo	F	R	Stroke	L	Frontal operculum, left insula	None	Anarthria, facial plegia, dysphagia, tongue paralysis	No improvement of anarthria at 12 years, slight improvement of dysphagia after few months
Starkstein et al. (1998) [7]	55 yo	M	R	Stroke	R	Frontal operculum and insula	None	Anarthria, dysphagia, jaw palsy	Improvement of jaw palsy and dysphagia, persistent anarthria
Moragas Garrido et al. (2007) [8]	61 yo	M		Stroke	L	Frontal operculum	None	Dysphagia, dysarthria, facial palsy	Absence of dysphagia and improvement of anarthria and palsy
	36 yo	M		Stroke	R	Frontal operculum	None	Dysarthria, dysphagia, facial diplegia, left hemiparesis	Improvement of dysarthria and dysphagia, persistent facial diplegia
Giraldo-Chica et al. (2010) [9]	76 yo	F	R	Stroke	R	Frontal operculum	None	Dysphagia and anarthria	Improvement of dysphagia and persistent anarthria at 18 months
Yildiz et al. (2010) [10]	75 yo	F	—	Stroke	R	Frontal operculum	Bilateral lacunar infarctions	Dysphagia, anarthria, mouth and tongue palsy	Slight improvement at 1 month
Nitta et al. (2013) [11]	20 yo	F	R	Contusion	R	Frontal anterior operculum, premotor area and association area	None	Masticatory diplegia, central facial diplegia, bilateral palate palsy, tongue palsy	Severe impairment after 18 months
Brandao et al. (2013) [12]	47 yo	F	R	Stroke	L	Frontal operculum, left insula	None	Anarthria, mouth and tongue paralysis, dysphagia, right hemiparesis	Global improvement at 4 years
Sà et al. (2014) [13]	76 yo	M	R	Stroke	L	Frontal operculum	Right cerebellar lesion	Anarthria and voluntary bilateral facial, pharyngeal, lingual and masticatory paralysis	No improvement after 1 month
Ohtomo et al. (2014) [14]	76 yo	M	R	Stroke	L	Frontal operculum	Left cerebellar and right ventral pontine	Anarthria, dysphagia, right hemiparesis, mouth palsy	Muscle paralysis resolved at 2 months, dysphagia improved at 5 months, no improvement of anarthria
Kim et al. (2015) [15]	—	—	—	Stroke	L	Frontal operculum, insula	Corona radiata	Anarthria	Complete recovery at 6 months
Shoji et al. (2019) [16]	84 yo	M	R	Abscess	L	Temporal operculum	Contralateral insular lesion	Dysphagia, anarthria, mouth and tongue palsy, mouth half open	1 week after abscess drainage ability to speak and swallow
Rivas et al. (2021) [1]	83 yo	F		Stroke	R	Frontal operculum	Leukoaraiosis	Dysarthria, left hemiparesis, dysphagia, not able to close the mouth	Partial improvement at 2 weeks

*Note:* H = handness, L = left, R = right, M = male, F = female.

Abbreviation: Yo = years old.

## Data Availability

The data that support the findings of this study are available from the corresponding author upon reasonable request.
